# Study on Improving Thickness Uniformity of Microfluidic Chip Mold in the Electroforming Process

**DOI:** 10.3390/mi7010007

**Published:** 2016-01-13

**Authors:** Liqun Du, Tong Yang, Ming Zhao, Yousheng Tao, Lei Luo, Lei Wang, Chong Liu

**Affiliations:** 1 Key Laboratory for Precision and Non-traditional Machining Technology of Ministry of Education, Dalian University of Technology, Dalian 116024, China; chongl@dlut.edu.cn; 2 Key Laboratory for Micro/Nano Technology and System of Liaoning Province, Dalian University of Technology, Dalian 116024, China; ariel@mail.dlut.edu.cn (T.Y.); zhaoming1990@mail.dlut.edu.cn (M.Z.); taoyousheng@mail.dlut.edu.cn (Y.S.T.); leiluo1990@mail.dlut.edu.cn (L.L.); 3 CapitalBio Corporation, Beijing 101111, China; wanglei@capitalbio.com

**Keywords:** microfluidic chip mold, micro-electroforming, uniformity, ultrasonic agitation, second cathode

## Abstract

Electroformed microfluidic chip mold faces the problem of uneven thickness, which decreases the dimensional accuracy of the mold, and increases the production cost. To fabricate a mold with uniform thickness, two methods are investigated. Firstly, experiments are carried out to study how the ultrasonic agitation affects the thickness uniformity of the mold. It is found that the thickness uniformity is maximally improved by about 30% after 2 h electroforming under 200 kHz and 500 W ultrasonic agitation. Secondly, adding a second cathode, a method suitable for long-time electroforming is studied by numerical simulation. The simulation results show that with a 4 mm width second cathode used, the thickness uniformity is improved by about 30% after 2 h of electroforming, and that with electroforming time extended, the thickness uniformity is improved more obviously. After 22 h electroforming, the thickness uniformity is increased by about 45%. Finally, by comparing two methods, the method of adding a second cathode is chosen, and a microfluidic chip mold is made with the help of a specially designed second cathode. The result shows that the thickness uniformity of the mold is increased by about 50%, which is in good agreement with the simulation results.

## 1. Introduction

The microfluidic chip is more and more widely used in the biomedical field. It has been applied to trace-substance detection in blood [1,2], infectious-disease-virus detection [3], and single-cell separation [4], *etc.* Consequently, the processing technology of the chips has received unprecedented attention. In addition, the high-performance mold plays a vital role to fabricate a perfect chip. The metal mold fabricated by micro-electroforming has become the first choice for mass production of the chips, due to its advantages of long service life, good surface quality and high dimensional accuracy. However, electroformed microdevices have the problem of uneven thickness, and until now, post-processing has been used to guarantee the dimensional accuracy and the surface quality [5]. For example, the thickness of the electroformed microfluidic chip mold fabricated by our research group is seriously uneven. Its thickness difference reaches 247.37 μm, and corresponding nonuniformity is 108.0%, which brings challenges to its post-processing. Hence, it is significant and meaningful to investigate a method to improve the thickness uniformity of the mold.

Until now, the thickness uniformity of the electroformed layer is improved by using a moving cathode, a rotating cathode [6] or an air-pressure agitation [7], adopting pulse-reversed current [8], adding an insulating shield [9] or a second cathode [10], and optimizing process parameters [11]. However, these existing methods, such as using a moving cathode, adopting pulse current and optimizing process parameters can only improve the thickness uniformity to a certain extent. They have been used during the fabrication of the microfluidic chip mold by our research group. Thus, it is necessary to find a new method to combine with these existing methods and to further improve the thickness uniformity of the mold. Some studies have shown that ultrasonic agitation can improve the surface quality of the electroformed layer [12,13], and that a second cathode can improve the thickness uniformity of the simple electroformed pattern, such as a rectangular channel [10]. However, there are few studies on improving the thickness uniformity of the electroformed microfluidic chip mold by using ultrasonic agitation or a second cathode. Hence, in this paper, the effect of ultrasonic agitation and second cathode on the thickness uniformity of the microfluidic chip mold is investigated to find a new method to fabricate a mold with uniform thickness.

Firstly, experiments are done to study the impact of ultrasonic parameters on the thickness uniformity of the microfluidic chip mold. Secondly, simulation is performed to investigate the effect of second cathode on the thickness uniformity. Finally, by comparing the effect of ultrasonic agitation and second cathode on the thickness uniformity, the method of adding a second cathode is chosen to fabricate the mold. Thus, based on the simulation results and electroforming equipment, a second cathode is designed, with which a microfluidic chip mold is manufactured.

## 2. Experimental Study on Effect of Ultrasonic Agitation on Thickness Uniformity of the Mold

### 2.1. Electroforming Experiment

**Figure 1:**
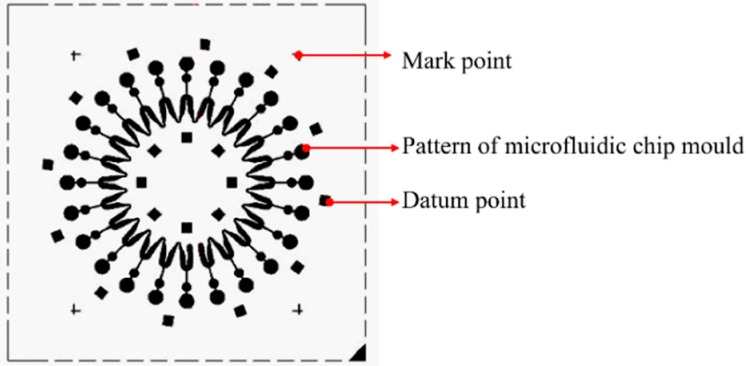
Photomask of the microfluidic chip mold.

Experimental procedures are as follows:

(1) Substrate treatment. Ni plates with the size of 63 mm × 63 mm × 5 mm are chosen as the substrates. Firstly, the substrates are ground and polished by precision lapping/polishing machine. Secondly, the substrates are washed with acetone and ethanol in sequence in ultrasonic cleaner for 15 min, respectively. Thirdly, the substrates are baked in an oven for 2 h at 85 °C.

(2) Fabrication of photoresist mold. SU-8 2075 photoresist (MicroChem Corp., Westborough, MA, USA) with a thickness of 100 μm is spincoated on the substrates and SU-8 photoresist molds are obtained after pre-bake, exposure, post-bake and development. The photomask used during the process of exposure is shown in Figure 1.

(3) Micro electroforming. The experimental parameters and electrolyte composition are shown in the reference [14]. The current density and electroforming time are 1 A/dm^2^ and 2 h, respectively. The electroforming experiments are divided into two groups to study the effect of the ultrasonic frequency *f* and power *P* on the thickness uniformity of the mold, respectively. In the first experimental group, *f* is set as 0, 80, 120 and 200 kHz, respectively, and the corresponding *P* is 200 W. In the second experimental group, *P* is set as 0, 100, 200 and 500 W, respectively, and the corresponding *f* is 200 kHz.

(4) Measurement. The blocks in Figure 1 are datum points, which are not electroformed during the electroforming process. On the basis of the datum points, the maximum and minimum thickness of the mold are measured by a surface profiler.

### 2.2. Results and Discussion

#### 2.2.1. Effect of Ultrasonic Frequency on Thickness Uniformity of the Mold

Nonuniformity α [11,15,16] is adopted to quantify thickness uniformity of the microfluidic chip mold.

(1) $$\alpha =\frac{h_{\max}-h_{\min}}{h_{\min}}\times 100\%$$

where, *h*_max_ and *h*_min_ stand for the maximum and minimum thickness of the mold, respectively.

Nonuniformity α of the mold fabricated under the different-frequency ultrasonic agitation are shown in Table 1. It is found that ultrasonic agitation reduces α of the mold, and that with ultrasonic frequency increasing from 80 to 200 kHz, α of the mold firstly increases and then decreases. With 80, 120 and 200 kHz ultrasonic agitation exerted, α of the mold is decreased by 25.8%, 16.7% and 29.2%, respectively.

**Table 1:** Dependence of α on ultrasonic frequency.

*f* (kHz)	*h*_max_ (μm)	*h*_min_ (μm)	α (%)
0	62.22	20.09	209.8
80	53.46	20.91	155.7
120	56.22	20.46	174.8
200	51.55	20.73	148.6

During the electroforming process, when the ultrasonic sound wave transmits to the sidewall of the photoresist mold, the wave amplitude is rapidly changed, causing a steady flow in the mold [17]. Additionally, during the alternating expansion and compression cycles of the ultrasonic sound waves, bubbles in the electrolytes expand and contract until collapse, forming a liquid jet or a pressure wave [18]. Through the two ways above, acoustic streaming and acoustic cavitation, ultrasonic agitation improves the mass transfer process, and then reduces the thickness of the diffusive layer, which can improve the thickness uniformity of the electroformed microstructure [15]. Hence, ultrasonic agitation can improve the thickness uniformity of the mold.

Additionally, the ultrasonic frequency has a great influence on the intensity of ultrasonic cavitation and acoustic streaming. With the ultrasonic frequency increasing, the ultrasonic cavitation intensity decreases because expansion and contraction time of bubbles is shortened, which leads to the difficulty of bubble collapse [19]. While the acoustic streaming intensity increases, acoustic streaming is largely confined to a thin viscous boundary layer, whose thickness is related to $f^{-\frac{1}{2}}$ [17], and the increase of the ultrasonic frequency leads to the decrease of the boundary layer thickness, which increases the acoustic streaming intensity [20].

Thus, with ultrasonic frequency increased from 80 to 120 kHz, ultrasonic cavitation intensity decreases, which weakens the effect of the ultrasonic agitation on the electrolyte. Consequently, the thickness uniformity of the mold decreases. With frequency further increased to 200 kHz, ultrasonic streaming intensity is increased, which strengthens the effect of the ultrasonic agitation on the electrolyte. Hence, the thickness uniformity increases. However, the effect of the ultrasonic frequency on the thickness uniformity of the mold increases in the order of 200 kHz > 80 kHz > 120 kHz > 0 kHz.

#### 2.2.2. Effect of Ultrasonic Power on Thickness Uniformity of the Mold

Nonuniformity α of the mold fabricated under different-power ultrasonic agitation is shown in Table 2. It is found that ultrasonic agitation reduces α of the mold, and that with ultrasonic power increasing from 100 to 500 W, α of the mold decreases. With 100, 200 and 500 W ultrasonic agitation exerted, α of the mold is decreased by 10.6%, 27.7% and 32.3%, respectively.

**Table 2:** Dependence of α on ultrasonic power.

*P* (W)	*h*_max_ (μm)	*h*_min_ (μm)	α (%)
0	61.92	20.08	208.3
100	60.86	21.26	186.3
200	54.39	21.71	150.5
500	53.47	22.19	141.0

The research has shown that with the increase of the ultrasonic power, acoustic streaming velocity [17] and ultrasonic cavitation intensity increase. Hence, the increase of the power strengthens the effect of the ultrasonic agitation on the electrolyte and improves the thickness uniformity of the mold.

In general, with ultrasonic agitation exerted during the electroforming process, the thickness uniformity of the microfluidic chip mold can be maximally improved by about 30%.

## 3. Simulation Study on Effect of a Second Cathode on Thickness Uniformity of the Mold

### 3.1. Geometric Model

**Figure 2:**
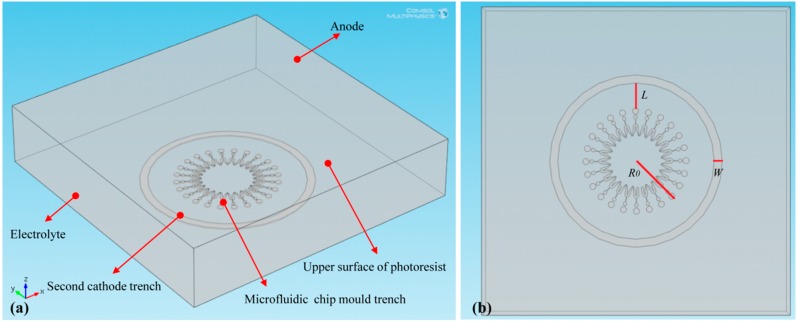
Electroforming geometric model. (**a**) pictorial drawing, (**b**) top view.

The simulation is performed by using the Electrochemistry Module of COMSOL Multiphysics software (COMSOL Inc., Stockholm, Sweden). In addition, a geometric model is established based on the following simplifications: (1) Second cathode is attached to the cathode, hence, with the aim of reducing the amount of calculation, it is assumed in the same plane with the cathode [21]. (2) The anode is the Ni plate, so it is simplified as a plane. (3) An assumption that the cathode consists of the upper surface of the photoresist, the trench of the microfluidic chip mold and the trench of the second cathode. The bottoms and sidewalls of the trenches are the electroformed areas and the photoresist, respectively. Therefore, the geometric model is shown in Figure 2a. *L* and *W* in Figure 2b, respectively, stand for the distance between the second cathode and the microstructure of the mold and the second cathode width. When *L* and *W* are both 0 mm, the second cathode is not present during the electroforming process. The simulation is divided into three groups. In the first group, *L* is set as 13, 9, 5 and 1 mm, respectively. In addition, the corresponding *W*, depth of the trenches *h* and electroforming time *t* are 4 mm, 100 μm and 2 h, respectively. In the second group, *W* is set as 4, 5, 6 and 7 mm, respectively. Additionally, the corresponding *L*, *h* and *t* are 9 mm, 100 μm and 2 h, respectively. In the third group, *t* is set as 2, 5, 10, 15 and 22 h, respectively. Additionally, the corresponding *L*, *W* and *h* are 9 mm, 4 mm and 500 μm, respectively.

### 3.2. Electrochemical Model

During the electroforming process, the electric field in the electrolyte is the following:

(2) $$\nabla \cdot i_{l}=Q_{l},i_{l}=-\sigma \nabla \phi_{l}$$

where *i_l_* is current density, *Q_l_* is general source term, σ is conductivity, and Ф*_l_* is potential.

With the polarization of the anode neglected, the anode is simplified to an isopotential surface. Hence, a potential of 0 V is added to the anode surface.

Boundary condition for the electroforming area is given as:

(3) $$i=-j\cdot \left\{S+\pi \left[{\left(R_{0}+L+W\right)}^{2}-{\left(R_{0}+L\right)}^{2}\right]\right\}$$

where *i* is total current, “–” means that electrons outflow from the electrode, *j* is current density of the cathodes (*j* = 1 A/dm^2^), *S* is surface area of the microfluidic chip mold (*S* = 473.04 mm^2^), and *R*_0_ is distance between the center point and the most outer point of the mold (*R*_0_ = 30 mm), as shown in Figure 2b.

A second cathode can improve the thickness uniformity of the mold by thieving some current from the mold during the electroforming process, hence, the influence of the mass transfer on the thickness uniformity is neglected. In addition, the Bulter-Volmer expression is used to describe the electrode reaction kinetics for the two cathode surface reaction:

(4) $$i_{\mathrm{ioc}}=i_{0}\left(\exp\left(\frac{\alpha_{a}F\eta }{RT}\right)-\exp\left(\frac{-\alpha_{c}F\eta }{RT}\right)\right)$$

where *i*_ioc_ is current density, *i*_0_ is exchange density, α*_a_* and α*_c_* are anode and cathode transfer coefficient, *F* is Faraday constant, *R* is universal gas constant, and *T* is temperature. In addition, η is overpotential and is defined by:

(5) $$\eta =\phi_{s}-\phi_{l}-E_{\mathrm{eq}}$$

where Ф*_s_* is potential of cathode surfaces, and *E*_eq_ is equilibrium potential and initial condition for the electroforming is:

(6) $$\phi_{s}=0\;V,\phi_{l}=0.257\;V$$

Based on Faraday’s law, the nickel depositing velocity can be deduced as:

(7) $$V_{\mathrm{dep}}=\frac{MN}{\rho }=-\frac{i_{\mathrm{ioc}}}{F}\frac{\nu M}{n\rho }$$

where *V*_dep_ is depositing velocity, *M* is molar mass, ρ is density, *N* is molar flux, ν is stoichiomic coefficient, and *n* is electron number of the reaction. The simulation parameters are shown in Table 3.

**Table 3:** Simulation Parameters.

σ (S/m)	*i*_0_ (A/m^2^)	α*_a_*	α*_c_*	*T* (K)	*E*_eq_ (V)	*M* (kg/mol)	ρ (kg/m^3^)	ν	*n*
0.95	17.1	1.5	0.5	324	−0.257	0.0586	8900	1	2

### 3.3. Results and Discussion

#### 3.3.1. Effect of Distance between Second Cathode and Microstructure of the Mold on Thickness Uniformity of the Mold

**Figure 3:**
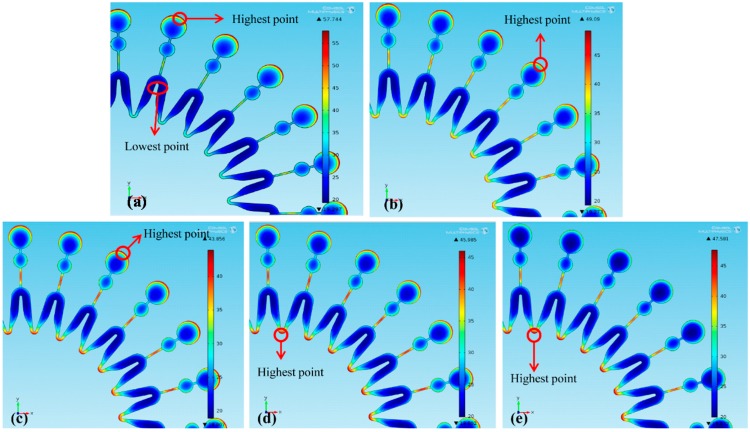
Thickness distribution of the mold: (**a**) without a second cathode; (**b**) *L* = 13 mm; (**c**) *L* = 9 mm; (**d**) *L* = 5 mm; (**e**) *L* = 1 mm.

Thickness distributions of the molds under different *L* are shown in Figure 3 (dimension unit is μm). Due to the central symmetrical mold with 24 same units, the thickness distributions in Figure 3 can sand for those of the whole mold. Figure 3a shows that without a second cathode exerted during the electroforming process, the maximum and minimum thickness points are respectively located at the edge of the big circle and the bend region of each unit. When a second cathode is exerted, with *L* increasing, the maximum thickness point gradually moves to the intersection between two units as shown in Figure 3b–e. Meanwhile, nonuniformity α are shown in Table 4. It is found that the thickness uniformity of the mold is improved by the second cathode, and α firstly decreases and then increases with *L* decreasing. With a second cathode of *L* = 13, 9, 5 and 1 mm used, the thickness uniformity of the mold is improved by 22.3%, 33.8%, 34.2% and 30.6%, respectively.

**Table 4:** Dependence of α on *L*.

(*L*, *W*) (mm)	*h*_max_ (μm)	*h*_min_ (μm)	α (%)
(0, 0)	57.74	19.30	199.3
(13, 4)	49.09	19.27	154.8
(9, 4)	43.86	18.91	131.9
(5, 4)	45.99	19.90	131.1
(1, 4)	47.58	19.97	138.3

**Figure 4:**
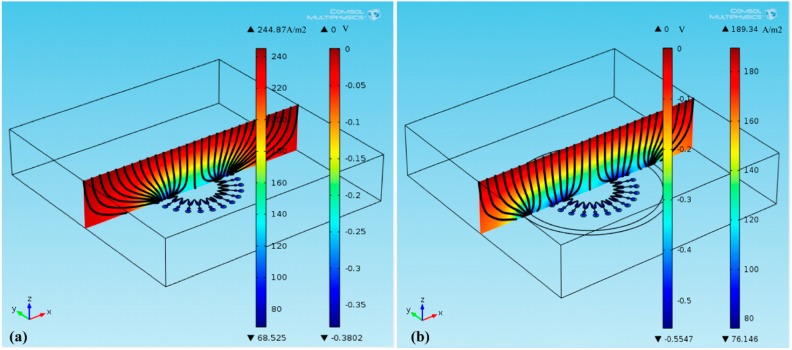
Current density distribution at the mold surface, and potential distribution and current-density streamline at the electrolyte cross-section: (**a**) without a second cathode; (**b**) with a second cathode.

Based on Faraday's law, the thickness of the electroforming layer is proportional to the current density at the electroformed layer surface, so the analysis for the thickness distribution of the mold can be regarded as the analysis of the current density distribution at the mold surface. Figure 4a,b respectively show the current density distribution at the mold surface, and the potential distribution and the current-density streamline at the electrolyte cross-section under the conditions of electroforming with and without a second cathode present. Black lines in the cross-section are current-density streamlines, and the denser the streamline is, the larger is the current density. In Figure 4a, it is found that without a second cathode present, the streamline at the edge of the mold’s big circle is denser, which means the current density there is larger. In addition, Figure 4b shows that with a second cathode exerted, some streamlines in the trench of the mold are stolen into the trench of the second cathode. Consequently the streamline at the edge of the big circle is reduced, which decreases current density there. Therefore, a second cathode can improve the thickness uniformity of the mold by thieving current.

**Figure 5:**
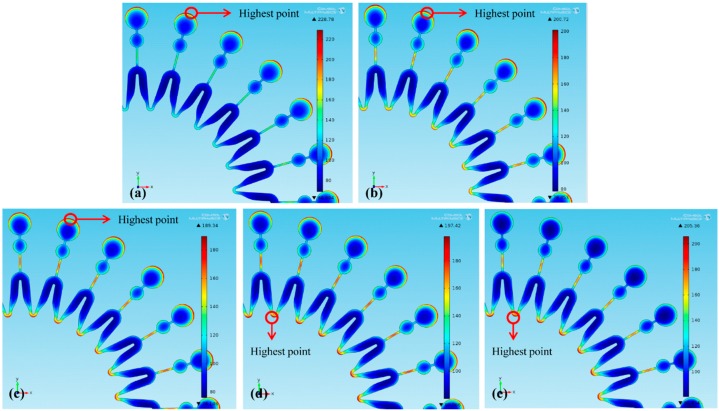
Current density distribution at the mold surface: (**a**) without a second cathode; (**b**) *L* = 13 mm; (**c**) *L* = 9 mm; (**d**) *L* = 5 mm; (**e**) *L* = 1 mm.

**Figure 6:**
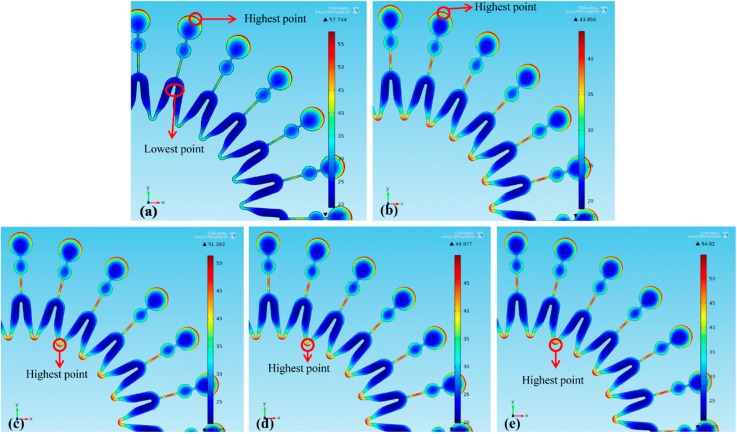
Thickness distribution of the electroforming mold: (**a**) without a second cathode; (**b**) *W* = 4 mm; (**c**) *W* = 5 mm; (**d**) *W* = 6 mm; (**e**) *W* = 7 mm.

Additionally, the current distributions at the mold surfaces are shown in Figure 5 (dimension unit is A/m^2^). Since smaller *L* makes more current stolen, with *L* reduced from 13 to 9 mm, more current at the edge of the mold’s big circle is stolen, which decreases the maximum current density from 200.72 to 189.34 A/m^2^, as shown in Figure 6b,c. Hence, the thickness uniformity of the mold is improved. With *L* further reduced to 5 mm, much more current stolen makes the intersection between two units become maximum current-density point, as shown in Figure 6d. And then with *L* further reduced to 1 mm, the current density at the intersection between two units is increased, and the thickness uniformity of the mold is reduced. Hence, with *L* increased, α firstly decreases and then increases.

#### 3.3.2. Effect of Second Cathode Width on Thickness Uniformity of the Mold

Thickness distributions of the molds under different *W* are shown in Figure 6 (dimension unit is μm). With *W* increasing, the maximum thickness point of the mold is firstly located at the edge of each unit’s big circle and then at the intersection between two units. In addition, nonuniformity α are shown in Table 5. It shows that the increasing *W* leads to the increase of α. With a second cathode of *W* = 4, 5, 6 and 7 mm used, the thickness uniformity of the mold is improved by 33.8%, 28.1%, 24.1% and 18.9%, respectively.

**Table 5:** Dependence of α on *W*.

(*L*, *W*) (mm)	*h*_max_ (μm)	*h*_min_ (μm)	α (%)
(0, 0)	57.74	19.29	199.3
(9, 4)	43.86	18.91	132.0
(9, 5)	51.29	21.08	143.3
(9, 6)	49.98	19.89	151.3
(9, 7)	54.82	20.95	161.7

**Figure 7:**
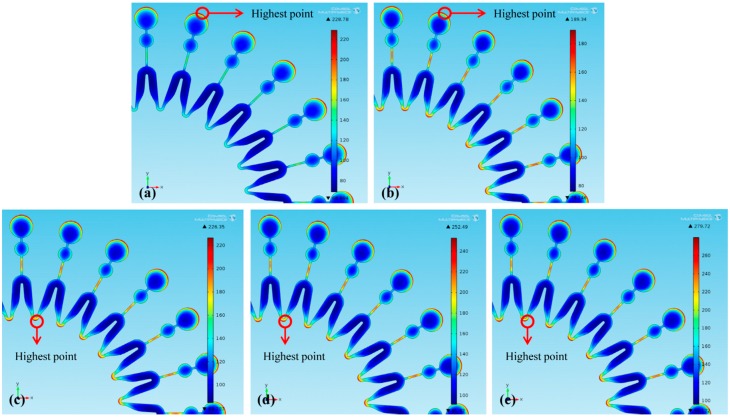
Current density distribution on the mold surface: (**a**) without a second cathode; (**b**) *W* = 4 mm; (**c**) *W* = 5 mm; (**d**) *W* = 6 mm; (**e**) *W* = 7 mm.

Current density distribution at the mold surfaces are shown in Figure 7 (dimension unit is A/m^2^). Since the larger *W* makes more current stolen by the second cathode, with *W* increased from 4 to 5 mm, the maximum current-density point moves to the intersection between two units from the edge of each unit’s large circle, and the maximum current density increases from 189.34 to 226.35 A/m^2^, as shown in Figure 7b,c. Consequently, the thickness uniformity of the mold decreases. With *W* further increasing to 6 mm and then to 7 mm, the current density at the intersection between two units gradually increases, which further reduces the thickness uniformity. Therefore, with *W* increasing, nonuniformity of the mold increases.

Generally, with a second cathode exerted, the thickness uniformity of the mold is maximally improved by about 30%.

#### 3.3.3. Effect of Electroforming Time on Thickness Uniformity of the Mold

Nonuniformity α under different electroforming times are shown in Table 6. It is found that α in the table is lower than those in Table 1 and Table 2 and Table 4 and Table 5, and that the thickness uniformity of the mold gradually decreases during the electroforming process. However, with a second cathode exerted, the effect of the second cathode on the thickness uniformity gradually becomes better. After 2, 5, 10, 15 and 22 h electroforming, the second cathode respectively improves the thickness uniformity by 31.2%, 33.1%, 35.2%, 38.4% and 45.5%.

**Table 6:** Dependence of α on electroforming time.

*t* (h)	2	5	10	15	22
α_without_ (%)	76.5	80.6	88.9	100	123.8
α_with_ (%)	52.6	53.9	57.6	61.6	67.5

Table 6 and other tables shows the nonuniformity of the mold deposited in 500 μm thickness photoresist mold and 100 μm thickness photoresist mold, respectively. The thicker photoresisit mold can better shield the electric field lines at the edge of the microstructures and offers the reactive ions more time to evenly diffuse to the cathode surface, which leads to a more uniform electroformed layer. Hence, α in Table 6 is lower than those in Table 1 and Table 2 and Table 4 and Table 5 [21]. Addtionally, during the electroforming process, nickel ion is firstly deposited on the cathode surface and then deposited on the formed electroformed layer. If the electroformed surface is uneven, since the anode is closer to the protruding part, where the current is easy to concentrate, the protruding part becomes more protruding. Consequently, the longer electroforming time is, the worse the thickness uniformity of the mold. Additionally, with a second cathode exerted, the second cathode improves the thickness uniformity of each electroformed layer and the electroformed layer deposited on an uniform electroformed layer is more uniform. Hence, with the electroforming time increasing, the effect of the second cathode on the thickness uniformity of the mold is better.

## 4. Fabrication of the Microfluidic Chip Mold

With ultrasonic agitation or a second cathode exerted during the electroforming process, the thickness uniformity of the microfluidic chip mold is maximally improved by about 30% after 2 h of electroforming. Considering it takes a long time to finish the electroformed mold with 230 μm thickness, the high temperature and pressure caused by ultrasonic agitation [22] may destroy the photoresist mold, influencing the quality of the mold. Hence, in this paper, the method of adding a second cathode is chosen to fabricate a microfluidic chip mold.

### 4.1. Fabrication of the Mold by Electroforming with a Second Cathode Present

**Figure 8:**
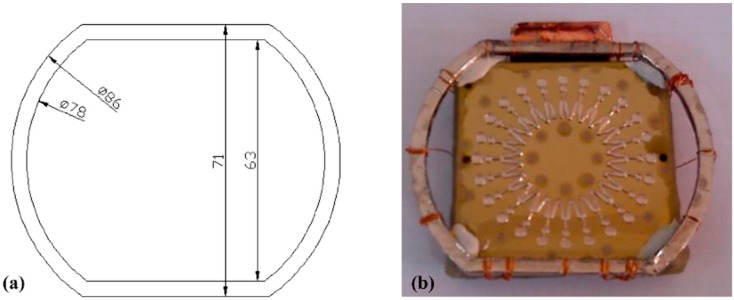
The second cathode: (**a**) design drawing; (**b**) physical prototype.

In order to investigate the effect of the designed second cathode on the thickness uniformity of the mold, a mold fabricated by electroforming without a second cathode present is used as a reference. According to the electroforming equipment and simulation results in Section 3.3.2, *L* and *W* are respectively set as 9 and 4 mm, and the second cathode is designed as Figure 8a (dimension unit is mm). The substrate with the second cathode is shown in Figure 8b.

**Figure 9:**
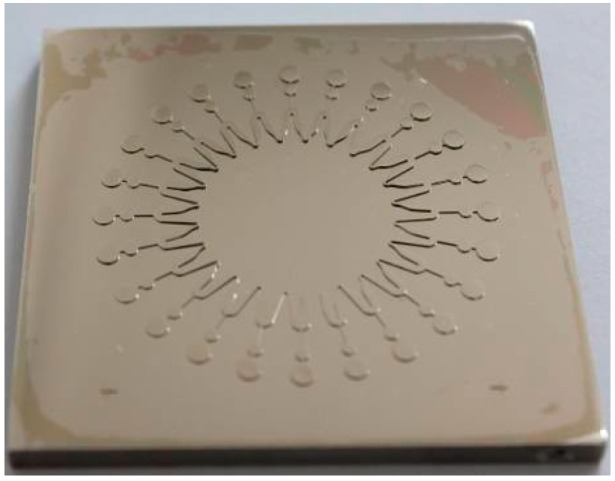
The microfluidic chip mold.

Ni plates with a size of 75 mm × 75 mm × 2 mm are chosen as the substrates. Firstly, SU-8 photoresist molds with a thickness of 230 μm are obtained after pre-bake, exposure, post-exposure bake and development. Secondly, the microfluidic chip molds are fabricated by 22-hour electroforming. At last, the photoresist molds are removed. The microfluidic chip mold is shown in Figure 9.

### 4.2. Uniformity Analysis of the Mold Thickness

Nonuniformity α is shown in Table 7. It shows that with the second cathode exerted, α of the mold decreases to 68.9% from 142.0%, and its thickness uniformity is improved by 51.5%. By comparing the results with the simulation results in Section 3.3.3, it is found that they are in good agreement. However, since the simulation neglects the effect of the mass transfer on the thickness uniformity of the mold, the uneven thickness of the photoresist mold and other influencing factors, the simulation result is a little smaller.

**Table 7:** Nonuniformity α of the microfluidic chip mold.

Electroforming Condition	*h*_max_ (μm)	*h*_min_ (μm)	α (%)
Without the Second Cathode	503.4	208.0	142.0
With the Second Cathode	344.8	204.1	68.9

## 5. Conclusions

In order to improve the thickness uniformity of the microfluidic chip mold, electroforming with the presence of ultrasonic agitation and a second cathode are respectively investigated. By comparing two methods, the method of adding a second cathode is chosen and a microfluidic chip mold is fabricated with the help of a specially designed second cathode. The conclusions are as follows:

(1) Ultrasonic agitation can improve the thickness uniformity of the microfluidic chip mold. With the increase of ultrasonic power, the thickness uniformity of the mold firstly increases and then decreases. In addition, with the increase of the ultrasonic frequency, the thickness uniformity increases. Additionally, the thickness uniformity is maximally improved by about 30% after 2 h of electroforming under 200 kHz and 500 W ultrasonic agitation.

(2) Electroforming with the presence of a second cathode can improve the thickness uniformity of the mold. When *W* is 4 mm, with *L* decreasing, the thickness uniformity of the mold firstly increases and then decreases. In addition, when *L* is 9 mm, with *W* increasing, the thickness uniformity decreases. The thickness uniformity is maximally improved by about 30% after 2 h electroforming under a second cathode whose *W* and *L* are 4 mm and 9 mm. Furthermore, when a second cathode is exerted, with electroforming time increasing, the improvement of the thickness uniformity gradually becomes better.

(3) According to the optimized simulation results and electroforming equipment, a second cathode is designed, with which a microfluidic chip mold is fabricated. The thickness uniformity of the mold is improved by about 50% after 22 h electroforming, which is in agreement with the simulation results. Hence, this simulation method can provide a reference for microelectroforming design.

(4) Due to the high temperature and pressure caused by the ultrasonic agitation, long-time electroforming may destroy photoresist mold; hence, this method is more suitable when the distance between cathodes and anodes is small. Electroforming with a second cathode present needs an extra electrode, and has no influence on electroforming environment. Hence, this method is more suitable for long-time electroforming. Additionally these two methods can all further improve the thickness uniformity of the microfluidic chip mold by combining with the existing methods.
